# Intelligence

**Published:** 1829-08

**Authors:** 


### INTELLIGENCE.

### MONTHLY REPORT OF DISEASES.

We have not observed any circumstances, in the general course of the diseases which have fallen under our notice since our last report, that merit particular detail. Upon the whole, the number of complaints has much diminished within the last three weeks, and those which have been most frequent have been slight, and easily controlled by ordinary treatment.

We have heard of a very severe and fatal fever at St. Alban's, that has prevailed there during the last month.

*Examination for Prizes and Honours at the University of London.*

We have only had an opportunity of looking over the answers given by one of the successful candidates, Mr. R. GARDNER, to the questions on Materia

1

Medica. They are many of them so excellent, so concise, yet so comprehensive, that we are induced to give them publicity.

The number of questions proposed by Professor THOMPSON was forty-five, all of which were answered by Mr. Gardner. We select the following, as being especially creditable both to the teacher and the taught. We were particularly struck with the neat and satisfactory answer given to the 21st question.

*Question* 3. What is white arsenic; by what chemical properties is it distinguished; and what are its effects on the system when it is taken into the stomach in an overdose?

*Answer.* White arsenic is an oxide possessing the properties of an acid, composed of one equivalent of metal and two equivalent of oxygen. It is distinguished by forming Scheele's green with the ammoniaco-sulphate of copper, a yellow precipitate with the ammoniaco-nitrate of silver, a white precipitate with lime-water, a yellow precipitate with sulphuretted hydrogen. Its effects as a poison are constriction in the throat, vomiting, purging, pain in the stomach and gripings, bloody stools, delirium, insensibility, with syncope and death. The body is said to run into very rapid putrefaction.

*Q.* 4. How is its presence in the stomach detected when it has been taken as a poison, and has proved fatal?

*A.* In the last answer the tests for white arsenic are enumerated: to satisfy a jury, I should boil the contents or substance of the stomach, filter the solution, pass sulphuretted hydrogen through it, collect the precipitate, dry it, and reduce to the metallic state by black flux.

*Q.* 9. What is the cause of the increased activity of gum-resins, possessing purgative properties, when they are combined with camphor?

*A.* Perhaps from camphor increasing the solubility of the gum-resin in the stomach. It may, however, be analogous to the increased power of purgatives when combined with tonics.

*Q.* 13. How, and from what part of the poppy is opium procured; what is its active principle; and what is the nature of its action on the animal economy?

*A.* Opium is obtained by wounding the unripe capsules in the evening, care being taken not to penetrate the seed-cells. In the morning the juice is scraped off, and inspissated by the heat of the sun. Its sedative principle is morphia, combined with meconic acid; its stimulating property is said to reside in narcotine. It acts directly on the nervous system, producing first stimulating effects, soon followed by collapse and sleep.

*Q.* 14. In what respect do the sedative effects of opium differ from those that follow the excitement of other stimulants on the animal system?

*A.* The sedative effects of opium are greater, in proportion to its stimulating effect, than the secondary effect of other stimulants. Its primary effect is very transient; when it produces its sedative effect, the mind is very tranquil. The secretion from the skin much augmented; the other secretions diminished.

*Q.* 17. Both astringents and stimulants influence the living system through the medium of the nerves. What is the most probable theory explanatory of their difference of effect on the system?

*A.* The effect of astringents is partly chemical, as is evident from their action on dead animal matter, and upon the mucous membrane of the mouth.

This, however, is probably combined with an action on the vital and nervous functions. But the last effect is more of a tonic nature, and not in so great a degree as the effect of stimulants. Stimulants produce their effects, when they stop hemorrhage, by stimulating the extreme vessels to contract.

*Q.* 21. What is the cause of the leaden hue which the internal administration of nitrate of silver sometimes communicates to the skin? Describe the manner in which it is effected, the changes that take place in the chemical composition of the nitrate, and in what part of the system these changes happen.

*A.* While the nitrate is in the circulation, it is under the control of the vital influence, but, when it gets to the skin, is subject to chemical affinities. The skin exhales muriate of soda and sulphuretted hydrogen. The effect of light is to blacken muriate of silver.

*Q.* 24. How does iodine affect the system? Is there any proof that it is conveyed into the blood?

*A.* Iodine acts particularly upon the glands. It passes into the blood, and may be detected by fecula in the urine of those who have taken it.

*Q.* 32. Why is the subacetate of lead more likely to produce colica pictonum than the acetate? And, in the event of its proving a poison, what antidote would you employ, and what principle would guide your selection of an antidote?

*A.* The subacetate is more likely to be precipitated as an oxide from its combination with a small quantity of acid; and we find that the carbonate and oxide are what generally produce the poisonous effects. I should give sulphates from sulphuric acid, forming an insoluble and inert compound with lead. Alum has been much recommended in colica pictonum. When lead acts as a poison, opium, castor oil, &c. may also be given.

*Q.* 35. In what manner does the tartrate of antimony and potassa produce its emetic effect?

*A.* I should suppose the emetic tartar acts by being taken into the circulation, and acting upon the nervous system through its medium. It produces much nausea. It acts as an emetic when injected into the blood, and when applied to a wound.

*Q.* 43. In a chemical point of view, in what does guaiacum differ from a gum-resin?

*A.* Resin is changed by concentrated acid to artificial tannin. Nitric acid changes guaiacum to oxalic acid. The sulphuric acid blackens it.

---

### BOTANY.

*Apothecaries' Hall; 26th June,* 1829.

Sir: I am directed by the Master and Wardens to transmit to you the following extract from the Minutes of a Court of Assistants of the Society of Apothecaries, held on Tuesday, the 23d instant.

I am, sir, your very humble servant,

John Sayer, Beadle.

"The Society of Apothecaries, anxious to promote the science of botany, and more particularly that branch of it which is immediately connected with the study and practice of medicine, have ordered that their Botanic Garden at Chelsea shall be opened on Friday, the 3d day of July, 1829, between the hours of nine and eleven; and on every succeeding Friday, at the same time, until further notice.

"It is intended that admission shall be given to all such medical students as are pupils to the established professors and tutors in the metropolis, whether in medicine, chemistry, materia medica, or botany. Such students to apply, at least three days prior, at the beadle's office in Apothecaries' Hall, for tickets of admission for that purpose; which the master and wardens will grant to such persons as they may think proper.

"In order that the master and wardens may be enabled to exercise suitable discretion in granting such tickets, each student must leave with the beadle a letter of recommendation from his tutor, stating that such student has been attentive to his studies, and is, in his opinion, desirous of improving himself in the science of medical botany.

"It is ordered that copies of these resolutions be forthwith sent to the respective professors and tutors in the branches of medical science above mentioned, containing a request that they will make the requisite communication to their pupils."

---

Dr. Prout.—This gentleman has been recently elected a Fellow of the College of Physicians, in conformity with what has lately become the custom of admitting a Licentiate annually to that honour.

---

*Ladies' Lying-in Institution.*—In a prospectus of this new speculation, the name of Mr. Houlton has been published as consulting surgeon. Mr. H. has addressed a letter to the Editor of the Medical Gazette, informing his medical friends that his name has been introduced through a *mistake*, and that he knew nothing of the *document* until he saw it, in an accidental manner, in print. If the directors of this extraordinary institution fall into many more such *mistakes*, the nature and policy of the society will be more accurately expressed by omitting the "*in*" in its denomination.

---

*Bloomsbury Dispensary.*—Mr. S. Cooper, author of the "Surgical Dictionary," &c. has been elected Surgeon to this institution by a very large majority of votes.

---

### MONTHLY LIST OF MEDICAL BOOKS.

### METEOROLOGICAL JOURNAL,

By Messrs. HARRIS and Co. Mathematical Instrument Makers, 50, High Holborn.

| June | Rain gauge. | Moon. | Thermom. | | | Barometer. | | De Luc's Hygrom. | | Winds. | | Atmospheric Variations. | | |
|---|---|---|---|---|---|---|---|---|---|---|---|---|---|---|
| | | | 9 A.M. | MAX. | MIN. | 9 A.M. | 10 P.M. | 9 A.M. | 10 P.M. | 9 A.M. | 10 P.M. | 9 *a.m.* | 2 *p.m.* | 10 *p.m.* |
| 20 | | | 70 | 72 | 54 | 29.77 | 29.69 | 40 | 40 | SSE | SE | Fine | Fine | Fine |
| 21 | | | 65 | 76 | 57 | .73 | .70 | 44 | 44 | ESE | SSE | —— | Cloudy | —— |
| 22 | | | 63 | 75 | 57 | .68 | .70 | 48 | 50 | SE | S | Rain | Show'ry | Fine |
| 23 | | | 68 | 77 | 60 | .80 | .85 | 50 | 49 | SS W | SSW | Fine | Show'ry | Cloudy |
| 24 | | ☾ | 70 | 74 | 59 | .92 | .96 | 47 | 45 | SS W | SW | Cloudy | Fine | Fine |
| 25 | | | 65 | 72 | 61 | .96 | .84 | 44 | 48 | S | ESE | Fine | —— | —— |
| 26 | .57 | | 68 | 73 | 58 | .84 | .73 | 48 | 50 | SSW | SW | —— | —— | —— |
| 27 | | | 64 | 68 | 59 | .57 | .36 | 50 | 52 | SW | ENE | Cloudy | Rain | Cloudy |
| 28 | .23 | | 65 | 70 | 55 | .43 | .49 | 53 | 55 | ENE | NE | Fine | Cloudy | Rain |
| 29 | | | 57 | 72 | 57 | .56 | .63 | 57 | 55 | N | NNW | Rain | Rain | Cloudy |
| 30 | .42 | | 67 | 67 | 56 | .63 | .62 | 55 | 56 | NW | WSW | Cloudy | Show'ry | Cloudy |
| July 1 | | ● | 57 | 68 | 54 | .52 | .35 | 57 | 56 | SSW | S | Rain | Rain | Rain |
| 2 | .60 | | 59 | 71 | 54 | .42 | .56 | 54 | 53 | SW | SWb.S | Fine | Fine | Fine |
| 3 | | | 62 | 69 | 52 | .48 | .26 | 52 | 51 | SWb.S | SWb.S | Cloudy | Fine | Cloudy |
| 4 | .14 | | 65 | 66 | 53 | .47 | .48 | 51 | 51 | WSW | WSW | Cloudy | Rain | Fine |
| 5 | | | 61 | 67 | 52 | .54 | .65 | 52 | 52 | SSW | WSW | Rain | Cloudy | Cloudy |
| 6 | | | 64 | 70 | 56 | .72 | .80 | 53 | 53 | W | W | Cloudy | Fine | Fine |
| 7 | | | 59 | 68 | 56 | .70 | .60 | 53 | 50 | WSW | SW | Rain | Rain | Cloudy |
| 8 | | | 63 | 71 | 57 | .69 | .63 | 50 | 50 | W | WSW | Fine | Fine | Fine |
| 9 | | ☽ | 61 | 67 | 50 | .67 | .74 | 48 | 46 | W | WNW | Fine | Show'ry | Fine |
| 10 | .60 | | 67 | 68 | 57 | .70 | .59 | 47 | 52 | W | SW | Fine | Fine | Show'ry |
| 11 | | | 60 | 64 | 56 | .47 | .32 | 54 | 54 | SW | SSW | Cloudy | Show'ry | Cloudy |
| 12 | | | 61 | 69 | 59 | .41 | .49 | 56 | 56 | SSW | SW | Show'ry | Show'ry | Rain |
| 13 | | | 64 | 70 | 62 | .56 | .67 | 57 | 57 | SW | SW | Cloudy | Fine | Fine |
| 14 | | | 68 | 74 | 59 | .75 | .72 | 57 | 54 | SW | WSW | Fine | Show'ry | Fine |
| 15 | .95 | | 68 | 74 | 59 | .85 | .82 | 54 | 50 | WSW | SW | Fine | Fine | Fine |
| 16 | | ○ | 67 | 69 | 57 | .82 | .75 | 47 | 50 | SW | WSW | Fine | —— | Cloudy |
| 17 | | | 64 | 65 | 58 | .72 | .47 | 50 | 55 | SW | SSW | Show'ry | Rain | Cloudy |
| 18 | | | 62 | 70 | 58 | .43 | .53 | 57 | 54 | SW | W | Cloudy | Rain | Rain |
| 19 | .61 | | 62 | 73 | 52 | .72 | .80 | 51 | 50 | W | W | Fine | Cloudy | Cloudy |

The quantity of Rain fallen in the month of June, was one inch and 41-100ths.

