# Erratum in the Last Number

**Published:** 1829-09

**Authors:** 


ERRATUM *in the last Number*, *p.* 185.—The name of the gentleman who gained the Materia Medica Prize, and other honours, at the University of London, is Mr. Robert Garner, of the Staffordshire Potteries, and not "Gardner," as it was misprinted.

